# Chronic Proliferative Dermatitis in *Sharpin* Null Mice: Development of an Autoinflammatory Disease in the Absence of B and T Lymphocytes and IL4/IL13 Signaling

**DOI:** 10.1371/journal.pone.0085666

**Published:** 2014-01-21

**Authors:** Christopher S. Potter, Zhe Wang, Kathleen A. Silva, Victoria E. Kennedy, Timothy M. Stearns, Lisa Burzenski, Leonard D. Shultz, Harm HogenEsch, John P. Sundberg

**Affiliations:** 1 The Jackson Laboratory, Bar Harbor, Maine, United States of America; 2 Department of Comparative Pathobiology, College of Veterinary Medicine, Purdue University, West Lafayette, Indiana, United States of America; University Hospital Hamburg-Eppendorf, Germany

## Abstract

SHARPIN is a key regulator of NFKB and integrin signaling. Mice lacking *Sharpin* develop a phenotype known as chronic proliferative dermatitis (CPDM), typified by progressive epidermal hyperplasia, apoptosis of keratinocytes, cutaneous and systemic eosinophilic inflammation, and hypoplasia of secondary lymphoid organs. *Rag1^−/−^* mice, which lack mature B and T cells, were crossed with *Sharpin^−/−^* mice to examine the role of lymphocytes in CDPM. Although inflammation in the lungs, liver, and joints was reduced in these double mutant mice, dermatitis was not reduced in the absence of functional lymphocytes, suggesting that lymphocytes are not primary drivers of the inflammation in the skin. Type 2 cytokine expression is increased in CPDM. In an attempt to reduce this aspect of the phenotype, *Il4ra^−/−^* mice, unresponsive to both IL4 and IL13, were crossed with *Sharpin^−/−^* mice. Double homozygous *Sharpin^−/−^*, *Il4ra^−/−^* mice developed an exacerbated granulocytic dermatitis, acute system inflammation, as well as hepatic necrosis and mineralization. High expression of CHI3L4, normally seen in CPDM skin, was abolished in *Sharpin^−/−^*, *Il4ra^−/−^* double mutant mice indicating the crucial role of IL4 and IL13 in the expression of this protein. Cutaneous eosinophilia persisted in *Sharpin^−/−^*, *Il4ra^−/−^* mice, although expression of *Il5* mRNA was reduced and the expression of *Ccl11* and *Ccl24* was completely abolished. TSLP and IL33 were both increased in the skin of *Sharpin^−/−^* mice and this was maintained in *Sharpin^−/−^*, *Il4ra^−/−^* mice suggesting a role for TSLP and IL33 in the eosinophilic dermatitis in SHARPIN-deficient mice. These studies indicate that cutaneous inflammation in SHARPIN-deficient mice is autoinflammatory in nature developing independently of B and T lymphocytes, while the systemic inflammation seen in CPDM has a strong lymphocyte-dependent component. Both the cutaneous and systemic inflammation is enhanced by loss of IL4 and IL13 signaling indicating that these cytokines normally play an anti-inflammatory role in SHARPIN-deficient mice.

## Introduction

SHARPIN was recently identified as a component of the linear ubiquitin chain assembly complex (LUBAC) which also contains RNF31 (previously HOIP) and RBCK1 (previously HOIL1) [1], [2], [3]. This ubiquitination complex is an important component of the NFKB signaling pathway which is a critical regulator of inflammation, the immune response, and lymphoid tissue development [2], [3], [4]. In addition, SHARPIN is a negative regulator of integrin beta 1 (ITGB1) [5], a component of cell adhesion and cell recognition in a variety of processes including embryogenesis, hemostasis, tissue repair, immune response, and tumor metastasis. The physiological importance of SHARPIN is evident in the complex phenotype seen in SHARPIN-deficient mice. A spontaneous mutation in exon 1 of *Sharpin* resulted in the chronic proliferative dermatitis (CPDM) mouse mutant (C57BL/KaLawRij-*Sharpin^cpdm^*/RijSunJ, hereafter referred to as *Sharpin^cpdm^*) [6], [7]. These mice develop systemic inflammation characterized by accumulation of eosinophils, macrophages, and neutrophils, most prominently in the skin, but also other tissues including the lung, liver, esophagus, and joints. Homozygotes have defective lymphoid organs characterized by absence of follicles and marginal zones in the spleen and absence of follicles in lymph nodes [8]. Peyer's patches are initially formed in neonatal mice, but regress after two weeks of age and are absent in adult mice [9]. Furthermore, SHARPIN-deficient mice have a defective T_H_1 immune response and a shift towards a T_H_2 response [10], [11].

The phenotype of *Sharpin^cpdm^* mice has striking similarities to autoinflammatory diseases in human patients with inflammatory skin disease. Prototypical autoinflammatory diseases such as TNF-receptor associated syndrome (TRAPS) and familial Mediterranean fever (FMF) are characterized by dermatitis, arthritis, serositis, and fever in the absence of evidence of a role for autoantibodies and self-reactive T cells [12]. Previous studies in *Sharpin^cpdm^* mice demonstrated that hematopoietic stem cells transferred into sublethally irradiated, wild-type recipients did not induce the CPDM phenotype, while reciprocal skin transplants maintained the donor phenotype suggesting that skin-intrinsic factors, rather than systemic effects, underlie the dermatitis [6], [13]. However, the precise role of lymphocytes in the development of the cutaneous and systemic inflammation has not been determined.

Eosinophils and macrophages are the predominant inflammatory cell types in the skin of CPDM mice [6]. This corresponds with an increased expression of type 2 cytokines and with an increase in chitinase-like proteins including CHI3L4 (chitinase 3-like 4), a hallmark of type 2 inflammatory responses [10], [14]. Depletion of IL5 following treatment with neutralizing anti-IL5 antibodies or crosses with IL5-deficient mice decreased the number of eosinophils, but did not ameliorate the dermatitis, suggesting a limited role for eosinophils in disease development [15]. On the other hand, systemic treatment with IL12 markedly reduced the inflammation, suggesting that suppression of type 2 cytokines is beneficial [10].

Here we report studies aimed at more precisely defining the role of lymphocytes and T_H_2 cytokines in CPDM inflammation. SHARPIN-deficient mice were crossed with mice deficient in recombination activating gene 1 (CByJ.Cg-*Rag1^tm1Mom^*, hereafter referred to as *Rag1^−/−^* mice) that lack mature B and T lymphocytes, and with IL4RA-deficient mice (BALB/c-*IL4ra^tm1Sz^/IL4ra^tm1Sz^*, hereafter *Il4ra* ^−/−^) that lack the functional IL4 and IL13 receptor. Most of the work defining the role of *Sharpin* in regulation of the NFKB and integrin pathways utilized the C57BL/KaLawRij-*Sharpin^cpdm^*/RijSunJ (*Sharpin^cpdm^*) allele; however, a second spontaneous allelic mutation, CBy.OcB3-*Sharpin^cpdm-Dem^*, has been defined but the specifics of its phenotype had not been characterized previously [7]. Comparison of the two strains shows similar inflammation in *Sharpin^cpdm-Dem^* mice but with a more rapid onset compared with *Sharpin^cpdm^* mice. Although mutations in both *Sharpin*-deficient strains are in exon 1, and result in truncation, strain-specific background modifying genes can also affect the phenotype, therefore we used the *Sharpin^cpdm-Dem^* mice for the crosses, as the genetic background closely matches that of the *Rag1^−/−^* and *Il4ra^−/−^* mice. The studies reported here show that a lack of lymphocytes did not affect the dermatitis, consistent with an autoinflammatory disease, but markedly attenuated the systemic inflammation. The absence of IL4 and IL13 signaling abrogated expression of the chitinase-like protein CHI3L4 and other type 2 associated chemokines, but resulted in more severe cutaneous and systemic inflammation. This indicates that IL4, IL13, or both act to suppress the inflammatory response in SHARPIN-deficient mice.

## Results

**Figure 1:**
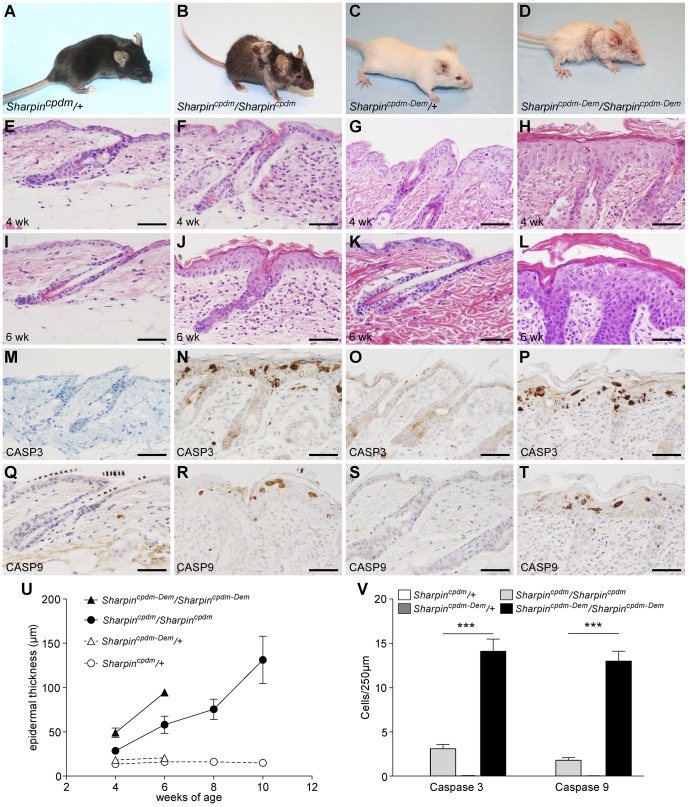
Mice with two different spontaneous mutant alleles of *Sharpin* show variations in onset of CPDM phenotype. At four weeks of age, mutant mice homozygous for the *Sharpin^cpdm^* (B) and *Sharpin^cpdm-Dem^* (D) alleles both exhibit a similar chronic proliferative dermatitis phenotype visible in H&E stained skin (*Sharpin^cpdm^* F,J; *Sharpin^cpdm-Dem^* H,L) when compared to WT littermates (E,I and G,K respectively). However, the onset is much more rapid in *Sharpin^cpdm-Dem^* mice as evidenced by the significantly increased epidermal thickening at four and six weeks (U) and by the increased numbers of apoptotic cells (v) indicated by antibody detection of cleaved CASPASE 3 (P) and CASPASE 9 (T) when compared to *Sharpin^cpdm^* mutants (N,R) or WT mice (M,Q,O,S). All data are means +/− SEM of N≥3. (***Significance indicated by P≤0.001).

The phenotype of *Sharpin^cpdm^* mice and their controls (**Fig. 1 A, B**) has been reported in detail [6], [8]. However, the *Sharpin^cpdm-Dem^* phenotype (**Fig. 1 C, D**) is less well characterized thus a detailed phenotypic comparison of the two alleles was performed. *Sharpin^cpdm-Dem^* mice have a phenotype similar to the original *Sharpin^cpdm^* mice, with severe dermatitis, systemic inflammation, and lymphoid organ defects. However, the skin and systemic inflammatory changes have a more rapid onset in *Sharpin^cpdm-Dem^* mice than in *Sharpin^cpdm^* mice as illustrated by shortened survival curves (**Fig. S1.**) and a significant increase (p<.001) in epidermal thickness and numbers of apoptotic keratinocytes by 4 weeks of age (**Fig. 1 U**). *Sharpin^cpdm^* mice usually require euthanasia by 10 weeks of age due to cutaneous ulceration, whereas *Sharpin^cpdm-Dem^* mice require euthanasia by 6–8 weeks of age. In addition, *Sharpin^cpdm-Dem^* mice have mild granulocytic inflammation of the adrenal medulla which was not observed in *Sharpin^cpdm^* mice (data not shown). To assess the role of T and B cells in the pathogenesis of the CPDM phenotype, *Sharpin^cpdm-Dem^* mice were crossed with *Rag1^−/−^* mutant mice, which lack mature B and T lymphocytes due to inactivation of the V(D)J recombination activation gene 1 (*Rag1*) [16]. A second independent cross was made using mice lacking the IL4 receptor alpha chain (*Il4ra*), a component of the receptor for IL4 and IL13, crossed with *Sharpin^cpdm-Dem^* mice. *IL4ra^−/−^* mice have a severely disrupted T_H_2 cytokine response due to their inability to respond to both IL4 and IL13 [17], [18]. In each case, age, and sex matched compound mutant mice were compared to *Sharpin^cpdm-Dem^, Rag1^−/−^*, or *Il4ra^−/−^* single gene mutants, and WT (+/+ or +/−) littermates as controls.

### Histopathology of Sharpin^cpdm-Dem^, Rag1^−/−^; Sharpin^cpdm-Dem^, Il4ra^−/−^, and control mice

#### Skin

**Figure 2:**
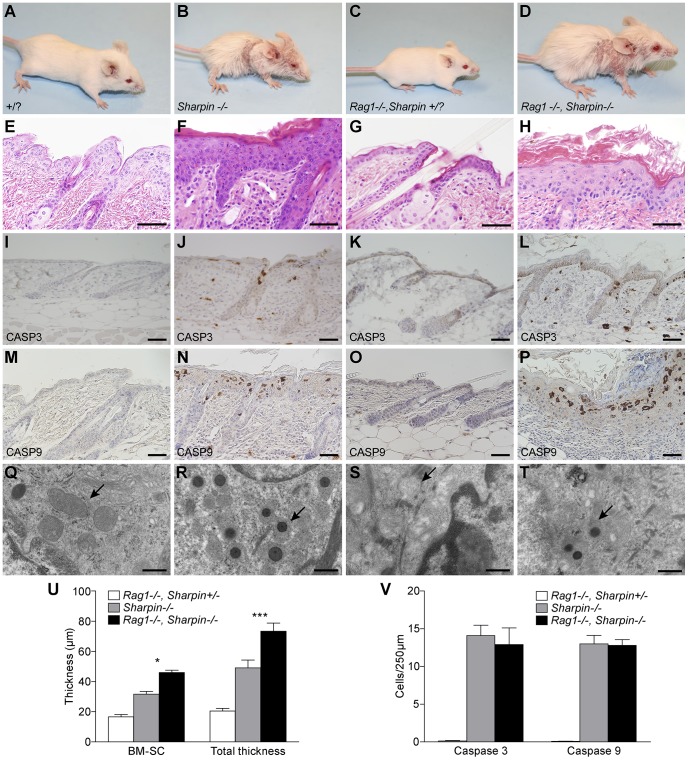
Dermatitis was not reduced in the absence of functional lymphocytes in *Sharpin^cpdm-Dem^, Rag1^−/−^* double mutants compared to *Sharpin^cpdm-Dem^* mice. Four week old *Sharpin^cpdm-Dem^, Rag1^−/−^* double mutants and *Sharpin^cpdm-Dem^* mice exhibit similar alopecia, puritis, and flaking skin (B,D) not seen in WT controls (a,c). H&E of skin from *Sharpin^cpdm-Dem^, Rag1^−/−^* double mutants (H) reveals epidermal hyperplasia with ortho- and parakeratotic hyperkeratosis and the mixed dermal inflammatory cell infiltrates typically seen in *Sharpin^cpdm-Dem^* mice (F) when compared to age matched WT controls (E,G). However, systematic measurement of the epidermal width reveals that it is a small but significant increase in total epidermal thickness (TE, basement membrane to top of the stratum corneum) and thickness of the Malphighian layer (basement membrane to the base of the stratum corneum (BM-SC) in double mutant mice compared to controls (U). However, *Sharpin^cpdm-Dem^, Rag1^−/−^* double mutants did not exhibit significantly increased numbers (V) of apoptotic keratinocytes as indicated by immunohistochemical detection of cleaved CASPASE 3 and 9 (J,N,L,P) when compared to controls (I,M,K,O). Examination by TEM reveals electron dense inclusions (arrows) in the mitochondria of epidermal keratinocytes in compound mutant skin (T) similar to those seen in *Sharpin^cpdm-Dem^* mice (R) which are absent in WT controls (Q,S). All data are means +/− SEM of N≥4. (Significance indicated by * and*** P≤0.001).

The dermatitis seen in *Sharpin^cpdm-Dem^* mice and *Sharpin^cpdm-Dem^, Rag1^−/−^* double mutants was similar in its severity (**Fig. 2 B,D**). Acanthosis, was present in both mutants with similar diffuse orthokeratotic hyperkeratosis and focal parakeratosis. Apoptotic keratinocytes were frequent within the epidermis and hair follicle root sheaths. Occasional granulocytes were present in the Malpighian layer and intracorneal and subcorneal pustules were seen in the upper epidermis (*p*≤0.001) (**Fig. 2 F,H**). The epidermis was significantly thicker in the *Sharpin^cpdm-Dem^, Rag1^−/−^* mutants when compared to *Sharpin^cpdm-Dem^* or control animals (**Fig. 2 U**). This exacerbation did not affect the frequency of apoptosis in epidermal keratinocytes (**Fig. 2 V**) as evidenced by similar numbers of cleaved CASP3/CASP9 positive cells (**Fig. 2 L,P**) when compared to *Sharpin^cpdm-Dem^* (**Fig. 2 j, n**) though both are higher in number than seen in WT controls (*p*≤0.001) (**Fig. 2 I, M and K,O**). Examination of skin from *Sharpin^cpdm-Dem^, Rag1^−/−^* mutants by transmission electron microscopy (TEM) also revealed mitochondrial inclusions similar to those previously described in *Sharpin^cpdm^* mice (**Fig. 2 Q, R, S, T**). There was a qualitatively similar accumulation of eosinophils, neutrophils, and mononuclear cells in the dermis in both mutants.

**Figure 3:**
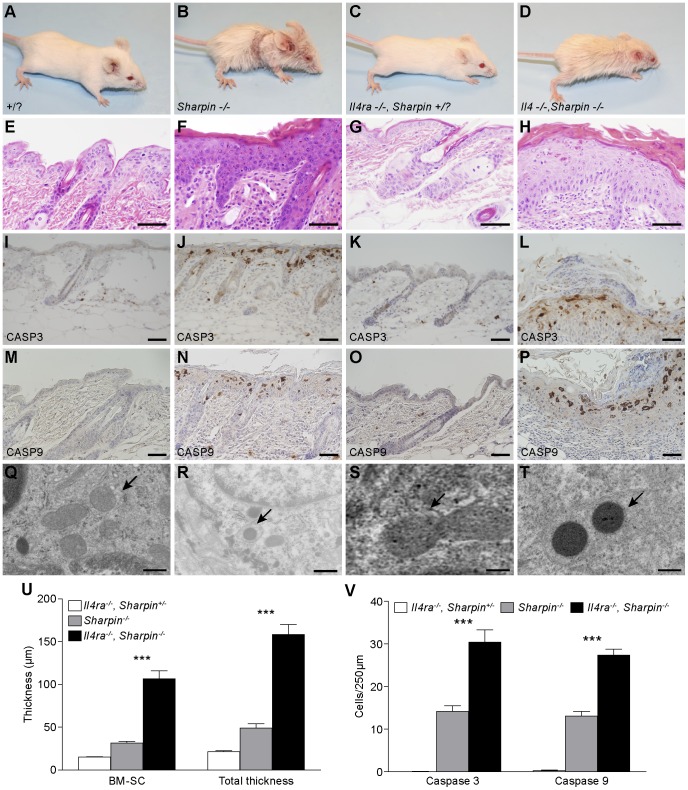
Loss of IL4RA signaling exacerbates the phenotype of *Sharpin* null mice. Four week old *Sharpin^cpdm-Dem^, Il4ra^−/−^* double mutants (D) exhibit a greatly exacerbated phenotype when compared to *Sharpin^cpdm-Dem^* mutants (B) or WT littermates (A,C). H&E of skin from *Sharpin^cpdm-Dem^, Il4ra ^−/−^* double mutants (H) reveals significantly increased epidermal hyperplasia with ortho- and parakeratotic hyperkeratosis when compared to *Sharpin^cpdm-Dem^* mice (F) or to *Il4ra* ^−/−^ (G) or WT (E) controls. Measurement of the epidermal width reveals that it is significantly increased in compound mutant mice compared to *Sharpin^cpdm-Dem^* and WT littermates both when measuring total epidermal thickness (TE, basement membrane to top of the stratum corneum) and thickness of the Malphighian layer (basement membrane to the base of the stratum corneum (BM-SC) (U). *Sharpin^cpdm-Dem^, Il4ra ^−/−^* double mutants also exhibited significantly increased numbers of apoptotic keratinocytes (V) as indicated by increased immunohistochemical detection of cleaved CASPASE 3 and 9 (J,L, and N,P respectively) when compared to controls (i,m,k,o). Examination by TEM reveals electron dense inclusions (arrows) in the mitochondria of epidermal keratinocytes of compound mutants, (t) similar to those seen in *Sharpin^cpdm-Dem^* mice (R) but absent in WT controls (Q,S). All data are means +/− SEM of N≥4. (Significance indicated by ***P≤0.001).

**Figure 4:**
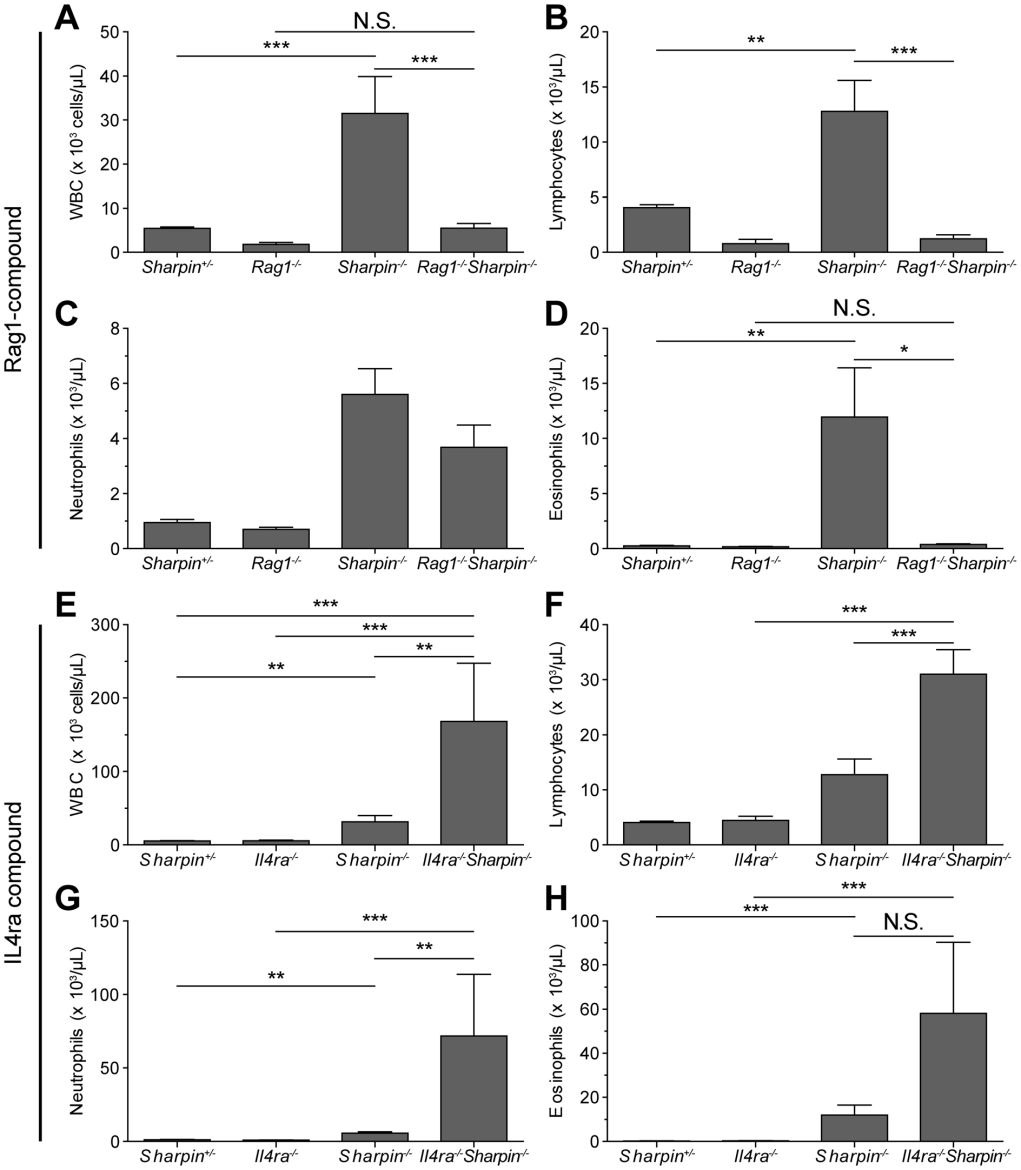
Peripheral blood analysis of *Sharpin^cpdm-Dem^, Rag1^−/−^* double mutant mice show a decline in systemic inflammation while *Sharpin^cpdm-Dem^, Il4ra^−/−^* double mutant mice reveals increased systemic inflammation compared to *Sharpin^cpdm-Dem^* and WT controls. Advia peripheral blood analysis reveals significant reductions in white blood cell counts (WBC) in peripheral blood of four week old *Sharpin^cpdm-Dem^*, *Rag1^−/−^* double mutant mice when compared to matched WT controls. This change is reflected in significant declines in eosinophils but not neutrophils. Differences in the number of lymphocytes reflect the lack of mature lymphocytes in *Rag1*null mice. White cell counts in peripheral blood of *Sharpin^cpdm-Dem^*, *Il4ra^−/−^* double mutant mice were greatly increased when compared to single mutant and WT controls. This change is reflected in significant increases in eosinophils, neutrophils, and lymphocytes. (Significance indicated by *P<0.05, ** P<0.01, and *** P<0.001).

The dermatitis in *Sharpin^cpdm-Dem^, Il4ra^−/−^* mice was much more severe than in the *Sharpin^cpdm-Dem^* mice (**Fig. 3 B, D**) and was associated with significantly increased epidermal thickening(*p*≤0.001) (**Fig. 3 U**) exceeding that in the *Sharpin^cpdm-Dem^, Rag1^−/−^* mutants, although in this case it was also accompanied by significantly greater numbers (*p*≤0.001) (**Fig. 3 V**) of cleaved CASP3 and 9 positive, apoptotic cells (**Fig. 3 L, P**) compared to *Sharpin^cpdm-Dem^* (**Fig. 3 J, N**) or WT controls (**Fig. 3 I, M, K, O**). As with both the *Sharpin^cpdm-Dem^* mice and *Sharpin^cpdm-Dem^, Rag1^−/−^* double mutants, TEM revealed abnormal inclusions in the mitochondria of epidermal keratinocytes that increase in number with disease progression (**Fig. 3 Q, R, S, T**). There was a significant increase in eosinophil numbers in the skin or peripheral blood of *Sharpin^cpdm-Dem^, Il4ra^−/−^* double mutants but it was not significantly higher than the elevations seen in *Sharpin^cpdm-Dem^* single mutant mice. However, overall white blood cell count (WBC) and neutrophil and lymphocyte counts were significantly increased in the peripheral blood of double mutants compared to single mutants or controls (*p*≤0.001) (**Fig. 4**).

#### Systemic inflammation

**Figure 5:**
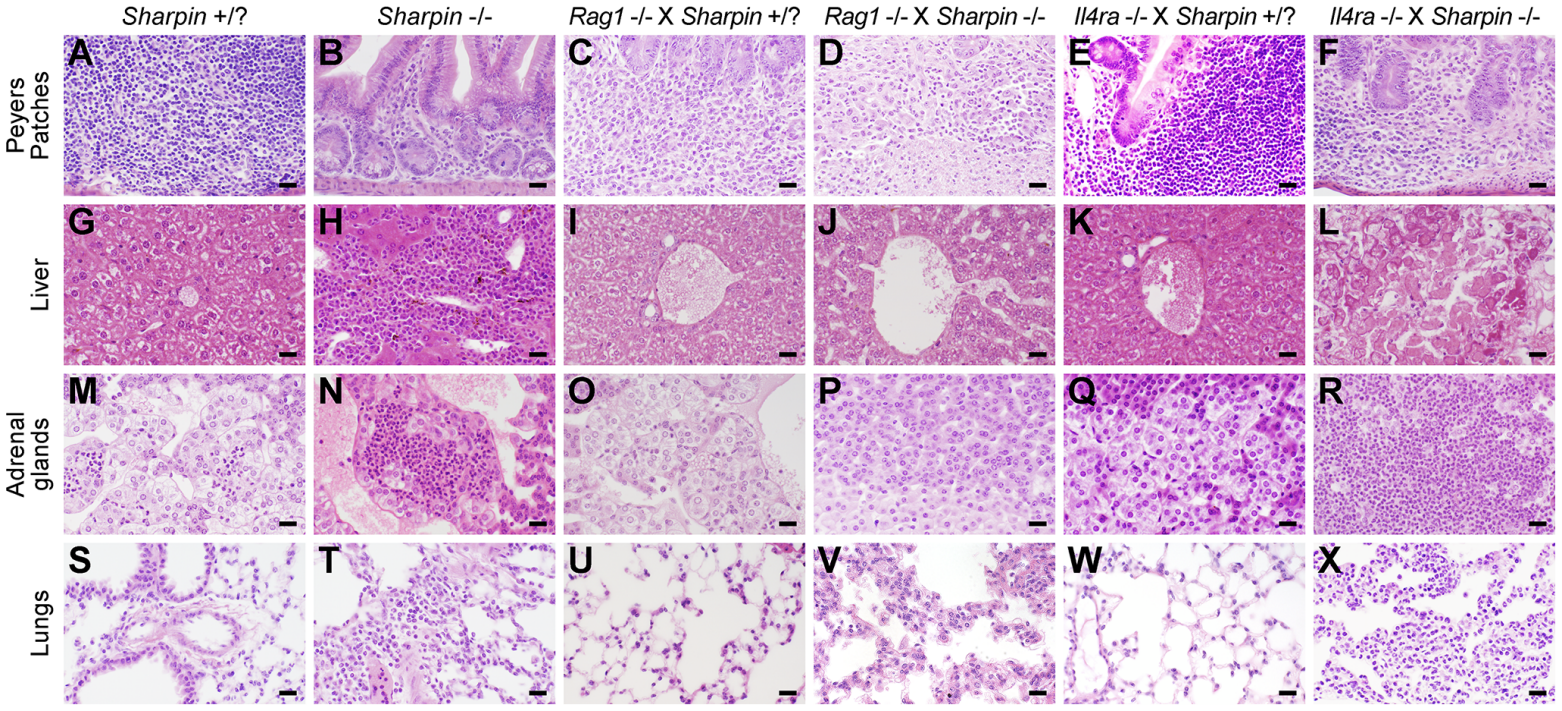
Systemic inflammation is decreased in *Sharpin^cpdm-Dem^*, *Rag1^−/−^* double mutant mice but significantly increased in *Sharpin^cpdm-Dem^*, *Il4ra^−/−^* double mutants compared to *Sharpin^cpdm-Dem^* and WT controls. Four representative histological comparisons of systemic inflammation in liver adrenal glands and lungs and lymphoid architecture in Peyer's patches in four week old WT (A,G,M,S), *Sharpin^cpdm-Dem^* (B,H,N,T); *Rag1^−/−^*(C,I,O,U); *Il4ra* ^−/−^ (E,K,Q,W); *Sharpin^cpdm-Dem^, Rag1^−/−^* (D,J,P,V); and *Sharpin^cpdm-Dem^, Il4ra* ^−/−^ (f,l,r,x) mice. Systemic inflammation is generally attenuated in *Sharpin^cpdm-Dem^, Rag1^−/−^* double mutant mice but exacerbated in *Sharpin^cpdm-Dem^*, *Il4ra* ^−/−^ double mutant mice compared to *Sharpin^cpdm-Dem^* controls.

*Sharpin^cpdm-Dem^* mice had moderate mixed cellular inflammation in portal triads and around the central vein in the liver, around bronchioles and blood vessels in the lung, and in the omentum in the peritoneal cavity, as previously described for *Sharpin^cpdm^* mice [6]. There was marked inflammation in the large joints (shoulder, knee, and elbow) and moderate inflammation in intervertebral joints. A few to moderate numbers of granulocytes were present in the adrenal medulla of the *Sharpin^cpdm-Dem^* mice as early as 22 days of age, which was not observed in the *Sharpin^cpdm^* mice. Inflammation was greatly attenuated in *Sharpin^cpdm-Dem^, Rag1^−/−^* mice with no or minimal inflammation present in the lungs, liver, or adrenal gland (**Fig. 5 D, J, P, V**), and mild to moderate inflammation in the joints. This reduction was reflected in the peripheral blood by significant reductions in numbers of WBC count and reduced percentages and numbers of eosinophils in peripheral blood (**Fig. 4 A**) although, as previously noted, this reduction in inflammation was not mirrored in the skin.

In marked contrast, inflammation was much more severe in *Sharpin^cpdm-Dem^, Il4ra^−/−^* double mutant mice than in *Sharpin^cpdm-Dem^* mice. There was extensive inflammation in the liver with focal to coalescing areas of coagulative necrosis and mineralization (**Fig. 5 L**). The adrenal medulla was nearly completely effaced by eosinophils (based on IHC staining for eosinophilic major basic protein, data not shown) (**Fig. 5 R**). There was also more extensive inflammation in the joints and peritoneal cavity than in *Sharpin^cpdm-Dem^* mice. Granulocytic inflammation in other tissues including the salivary glands, meninges, and colon, was present in the *Sharpin^cpdm-Dem^, Il4ra^−/−^* double mutant mice which was not observed in either of the *Sharpin* allelic mutations. The increased inflammation was reflected in the peripheral blood in the form of significant increases in WBC counts, lymphocytes, eosinophils, and neutrophils (**Fig. 4 E, F, G, H**).

### Cutaneous cytokine expression in *Sharpin^cpdm-Dem^, Il4ra^−/−^* mice

**Figure 6:**
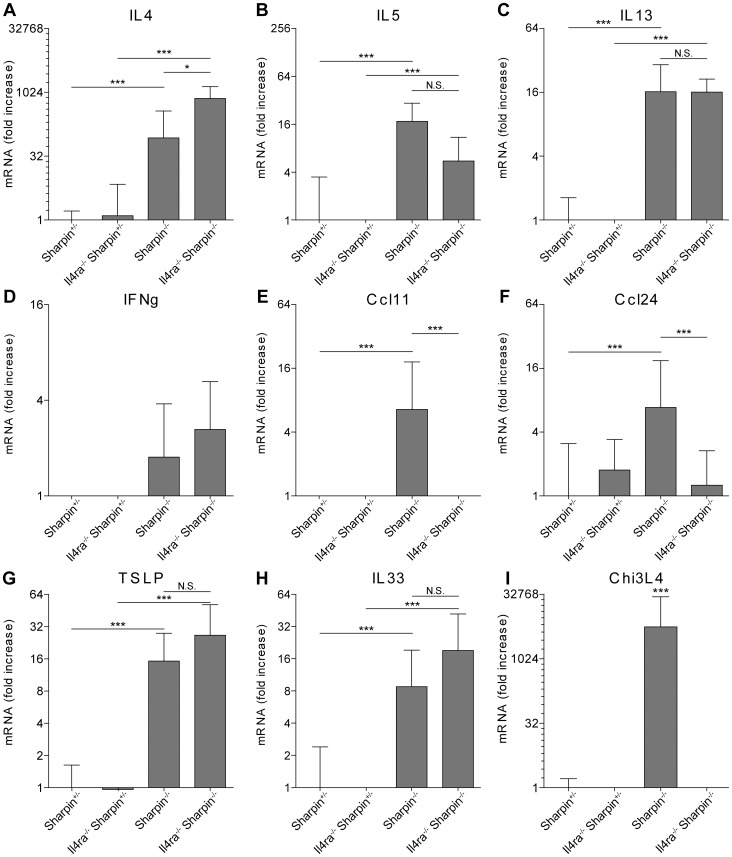
Quantitative RT-PCR reveals significant changes in cytokine expression in *Sharpin^cpdm-Dem^, Il4ra^−/−^* double mutant mice compared to single mutant and WT controls. RT-PCR was conducted on total RNA from four week old mouse skin taken from *Sharpin^cpdm-Dem^*, *Rag1^−/−^*; *Il4ra* ^−/−^; *Sharpin^cpdm-Dem^*, *Rag1^−/−^*; and *Sharpin^cpdm-Dem^*, *Il4ra* ^−/−^ mice (Significance indicated by * P<0.05, ** P<0.01, and *** P<0.001).

To determine the effect of loss of IL4 signaling on the expression of type 1 and type 2 cytokines in *Sharpin* null mice, changes in various cytokine mRNA levels were evaluated between WT; *Sharpin^cpdm-Dem^*; *Il4ra^−/−^*; and *Sharpin^cpdm-Dem^, Il4ra^−/−^* double mutant mice. Total RNA was isolated from the skin of mice at 4 weeks of age and tested by qRT-PCR. As reported previously [10], expression of *Il4*, *Il5*, and *Il13* mRNA was significantly increased in the skin of *Sharpin^cpdm-Dem^* mice (**Fig. 6 A, B, C**). In compound *Sharpin^cpdm-Dem^, Il4ra^−/−^* mice, *Il4* expression was greatly increased when compared with *Sharpin^cpdm^* mice or WT controls while the expression of *Il13* mRNA was still elevated but not significantly more than in *Sharpin^cpdm-Dem^* mice. Expression of *Ifng* transcripts was not significantly increased in the skin from *Sharpin^cpdm^* mice and *Sharpin^cpdm-Dem^, Il4ra^−/−^* mice (**Fig. 6 D**) suggesting that loss of IL4RA signaling in *Sharpin* null mice did not skew the phenotype towards production of type 1 cytokines.

In the absence of IL4 or IL13 in the *Sharpin^cpdm-Dem^, Il4ra^−/−^* mice, the expression of IL5 decreased (**Fig. 6 B**), but not enough to reach significance when compared to with *Sharpin^cpdm^* mice. CCL 11 (eotaxin) and CCL24 (eotaxin-2) are important eosinophil-specific chemokines induced by IL4 and IL13 [19]. We previously documented increased expression of CCL11 in the skin of *Sharpin^cpdm^* mice [20]. Here the expression of CCL11 and CCL24 was assessed in *Sharpin^cpdm-Dem^, Il4ra^−/−^* mice to determine if expression is affected by the absence of IL4 and IL13 signaling. The expression of both chemokines increased in the skin of *Sharpin^cpdm-Dem^* mice, (p<0.001), was completely abrogated in *Sharpin^cpdm-Dem^, Il4ra^−/−^* mice (**Fig. 6 E, F**) suggesting that the hypereosinophilia was not dependent on IL4, IL13, or on IL4 and IL13-induced chemokine expression in skin.

IL33 and TSLP, secreted by keratinocytes, promote a type 2 inflammatory response in skin [21], [22] RT-PCR indicated that the expression of TSLP and IL33 mRNA was significantly increased in *Sharpin^cpdm-Dem^* mice (p<0.001) (**Fig. 6**.) and that this upregulation was maintained in *Sharpin^cpdm-Dem^, Il4ra^−/−^* double mutant mice providing a possible explanation for the inflammation seen in both mutants.

The presence of alternatively activated macrophages is a hallmark of type 2 inflammation. One of the molecules typically expressed by these macrophages is the chitinase-like protein CHI3L4 [23] Increased expression of *Chi3l4* mRNA was previously observed in the skin of *Sharpin^cpdm^* mice [14] and was similarly observed in *Sharpin^cpdm-Dem^* mice (**Fig. 6 I**). However, this was completely abolished in *Sharpin^cpdm-Dem^, Il4ra^−/−^* mice (**Fig. 6 I**), indicating that IL4 and IL13 are critical for the induction of this protein.

## Discussion

Two independent spontaneous mutations in exon 1 of the *Sharpin* gene arose on two different inbred mouse strains, both resulting in the complex phenotype of chronic proliferative dermatitis (CPDM) mice. The *Sharpin^cpdm^* allelic mutant mice have a single base-pair deletion which occurred in mice on a C57BL/KaLawRij background, a strain closely related to C57BL/6J based on skin graft histocompatibility [7] whereas *Sharpin^cpdm-Dem^* mice have a 14 base pair deletion and a single base pair substitution. This latter allelic mutation is now maintained on the BALB/cByJ congenic background [7]. Although the phenotypes of the two strains of SHARPIN-deficient mice are qualitatively similar, the development of the skin lesions and systemic inflammation was accelerated in the *Sharpin^cpdm-Dem^* mice resulting in a much more severe phenotype at four weeks of age. The *Sharpin* mutations in both strains result in a premature stop codon in exon 1 and complete loss of SHARPIN expression [7]. This indicates that the phenotypic differences are not caused by residual expression of part of the SHARPIN protein, but rather by differences in strain-specific genetic polymorphisms that affect the inflammatory process. Because the *Rag1^−/−^* and *Il4ra^−/−^* mice were on a BALB/cJ or closely related BALB/cByJ congenic background, crosses were set up with *Sharpin^cpdm-Dem^* mice.

Loss of lymphocytes in *Sharpin^cpdm-Dem^, Rag1^−/−^* mice did not diminish the dermatitis consistent with the failure to induce the CPDM phenotype by hematopoietic cell transfer [6]. However, the systemic inflammation was markedly attenuated in the absence of lymphocytes. This demonstrates that lymphocytes are not necessary for the development of the cutaneous inflammation seen in CPDM and suggests that the skin lesions are autoinflammatory in nature. However, the lymphocyte dependence of the systemic inflammation also suggests that this phenotype has an autoimmune component. Autoinflammatory diseases are caused by activation of innate immune cells by endogenous or exogenous stimuli in the absence of autoantibodies and self-reactive T cells. Dermatitis is a common manifestation of autoinflammatory diseases in human beings in particular those that are associated with overexpression of IL1B (inflammasomopathies) and with defects in the NFKB signaling pathways [12], [24]. In the spectrum of autoimmune, rheumatoid, and autoinflammatory diseases, a mixture of autoinflammatory and autoimmune responses is probably not uncommon, and the inflammation in the SHARPIN-deficient mice is an example of such a complex pathogenesis. The inciting event that leads to the cutaneous inflammation in SHARPIN-deficient mice is not known. Mice with epidermal specific deletions of IKK2 develop severe inflammatory skin disease in a TNF-dependent manner, suggesting that NFKB signaling is a critical component [25]. Likewise increased keratinocyte apoptosis may contribute to inflammation, and is also tied to NFKB, as supported by the increased sensitivity of SHARPIN-deficient cells to TNF-induced necroptosis and the lack of dermatitis in TNF-deficient *Sharpin^cpdm^* mice [1]. Keratinocyte cell death may lead to the release of pro-inflammatory danger signals such as IL33, the expression of which was increased in the skin of the mutant mice. Keratinocyte-specific deletion of caspase 8 and the FAS-associated death domain (FADD) adaptor protein result in increased necroptosis of keratinocytes and cutaneous inflammation that is strikingly similar to the dermatitis in SHARPIN-deficient mice [26], [27], [28]. The inflammation in FADD-deficient mice is not affected by the lack of lymphocytes consistent with the autoinflammatory nature of the skin lesions [28]. FADD-deficient keratinocytes had increased sensitivity to TNF-induced cell death, and the development of the inflammation was delayed in TNFR1- and TNF-deficient FADD-deficient mice [28]. The dermatitis in mice with keratinocyte-specific deletion of caspase 8 (*Casp8^tm1Wll^*) is characterized by accumulation of eosinophils and macrophages and increased expression of type 2 cytokines similar to *Sharpin^cpdm^* mice [26], [27]. However, the dermatitis in CASP8-deficient mice is not affected by absence of TNF or IL1-receptor signaling [26], in contrast to *Sharpin^cpdm^* mice [1], [29]. This suggests that the pathogenesis of the cutaneous inflammation in SHARPIN-deficient mice is different from that in CASP8 and FADD-deficient mice and illustrates how different molecular pathways can lead to remarkably similar phenotypes. Upregulation of CASP9 expression seen in the skin of SHARPIN deficient mice could suggest an alternative, intrinsic apoptotic mechanism.

The cutaneous inflammation in SHARPIN-deficient mice is characterized by accumulation of eosinophils and overexpression of type 2 cytokines. Previous work showed that this dermatitis was not affected by depletion of IL5 and loss of eosinophils, but the inflammation could be prevented by systemic administration of IL12 [10], [15]. To further examine the role of type 2 cytokines, SHARPIN-deficient mice were crossed with *Il4ra^−/−^* mice, which abolished the function of IL4 and IL13. This effectively eliminated the expression of *Chi3l4* mRNA in the skin consistent with the induction of these proteins in macrophages and mast cells by IL4 and IL13 [14], [30], [31]. In addition, *Il4ra* deletion decreased the expression of *Il5*, though not to significant levels and abrogated expression of the eosinophil-specific chemokines CCL11 and CCL24. In spite of these changes, the dermatitis in *Sharpin^cpdm-Dem^*, *Il4ra^−/−^* CPDM mice was more severe with similar numbers of eosinophils. This indicates that other chemotactic factors are responsible for the recruitment of eosinophils into the skin of the double mutant mice. These findings are reminiscent of those in NC/Nga mice that were crossed with STAT6-deficient mice [32]. NC/Nga mice develop dermatitis with eosinophils and mast cells when housed in a conventional environment, usually associated with ectoparasite infestations, more a form of atopic dermatitis [33]. STAT6 is a transcription factor, pivotal for IL4 and IL13 signaling, and STAT6-deficient mice fail to develop a T_H_2 cytokine responses [34]. STAT6-deficient NC/Nga mice had similar skin lesions as intact NC/Nga mice with eosinophils and mast cells [32]. However, the authors reported that expression of CCL11 and CCL24 was not affected by the STAT6 deficiency.

The exacerbated phenotype seen in *Sharpin^cpdm-Dem^, Il4ra^−/−^* mice was associated with increased apoptosis of keratinocytes as well as degeneration and necrosis of hepatocytes. This may be attributed to the anti-apoptotic role of IL4 signalling [35]. Overexpression of IL4 in mouse keratinocytes prevented UVB-irradiation induced apoptosis, and *in vitro* treatment of normal human epithelial cells with IL4 induced the expression of anti-apoptotic proteins and reduced apoptosis by cytotoxic drugs [36], [37]. Increased apoptosis of keratinocytes in *Sharpin^cpdm-Dem^, Il4ra^−/−^* mice may lead to greater release of pro-inflammatory danger molecules and increased dermatitis. Hepatocytes of *Sharpin^cpdm^* mice had increased sensitivity to TNF-induced apoptosis [38], although degenerative changes and necrosis of hepatocytes are not features of the phenotype of *Sharpin^cpdm^* and *Sharpin^cpdm-Dem^* mice. However, in the absence of IL4RA-signaling extensive hepatic coagulative necrosis and mineralization was present, suggesting a greatly enhanced sensitivity of hepatocytes to TNF. IL4 has direct anti-inflammatory effects by inhibiting the secretion of pro-inflammatory cytokines by macrophages [39], [40]. A recent study demonstrated that IL4 induces the differentiation of newly recruited monocytes into anti-inflammatory M2 macrophages in a mouse model of allergic skin disease [41]. The increased expression of CHI3L3/4 proteins in the skin of *Sharpin^cpdm^* mice is consistent with an M2 phenotype of macrophages [14], and the absence of these CHI3L3/4 in the skin of IL4RA-deficient *Sharpin^cpdm^* indicates a lack of anti-inflammatory macrophages. Taken together, the exacerbated inflammation in IL4RA-deficient *Sharpin^cpdm-Dem^* mice can be attributed to increased apoptosis of keratinocytes and abrogation of the anti-inflammatory effect of IL4.

What may account for the accumulation of eosinophils and mast cells in the skin of IL4RA-deficient *Sharpin^cpdm-Dem^* mice? A possible explanation is the increased expression of the cytokines TSLP and IL33 in the skin of SHARPIN-deficient mice which was maintained in the absence of IL4RA. The expression of TSLP is increased in keratinocytes of patients with atopic dermatitis and keratinocyte-specific expression of TSLP induces atopic dermatitis-like inflammation in the skin of mice [42], [43]. TSLP also plays a critical role in a model of atopic dermatitis induced by topical administration of calcipotriol that is independent of T lymphocytes [44]. IL33 is a member of the IL1 family of cytokines and has been identified as an important factor in allergic diseases [45]. It can recruit and activate eosinophils directly. The expression of IL33 is increased in human patients with atopic dermatitis and in mouse models of atopic dermatitis [46]. The IL33 receptor is comprised of ST2 and IL1 receptor associated protein (IL1RAP). A role for IL33 in the dermatitis in SHARPIN-deficient mice is supported by the attenuation of inflammation in *Sharpin^cpdm^* mice in which IL1RAP was deleted [29]. However, this needs confirmation as IL1RAP is shared with other IL1-family receptors.

In conclusion, these studies determined that the cutaneous inflammation in SHARPIN-deficient mice develops independently of B and T lymphocytes and is autoinflammatory in nature, whereas the systemic inflammation has a strong lymphocyte-dependent component. Both cutaneous and systemic inflammation are enhanced by loss of IL4 and IL13 signaling indicating that this signaling pathway plays an anti-inflammatory role in SHARPIN-deficient mice.

## Materials and Methods

### Generation and care of mice

Stocks of C57BL/KaLawRij-*Sharpin^cpdm^*/RijSunJ; C.OcB3-*Sharpin^cpdm-Dem^/Sharpin^cpdm-Dem^*; CByJ.Cg-*Rag1^tm1Mom^*, *Sharpin^cpdm-Dem^*/Sz; and C.Cg-Il4ratm1Sz, *Sharpin^cpdm-Dem^*/Sz mice (The Jackson Laboratory; Bar Harbor, ME) were maintained in the humidity, temperature, and light cycle (12∶12) controlled vivarium under specific pathogen-free conditions (http://jaxmice.jax.org/genetichealth/health_program.html). Mice were housed in double-pen polycarbonate cages (330 cm^2^ floor area) at a maximum capacity of four mice per pen. Mice were allowed free access to autoclaved food (NIH 31, 6% fat; LabDiet 5K52, Purina Mills, St. Louis, MO) and acidified water (pH 2.8–3.2). All work was done with the approval of The Jackson Laboratory Animal Care and Use Committee under approval number 07005.

A second spontaneous *Sharpin* allelic mutation occurred on the OcB3/Dem inbred strain at the Institute of Molecular Genetics, Academy of Sciences, Czech Republic. This allele was moved on the BALB/cByJ strain to create the congenic CBy.OcB3-*Sharpin^cpdm-Dem^* line. These mice and their BALB/cByJ controls are also maintained at The Jackson Laboratory. The colony was maintained by heterozygous matings and genotyped using the primers D15Mit28. L: ATACACACGCACCCCCATAT; R: CACCACTGACCAATGAGCC [7]. *Rag1^−/−^* mice were maintained as homozygotes as previously described [16] and were genotyped by PCR using primers: 5′ - TGGATGTGGAATGTG TGCGAG –Mutant Forward; 5′ - GAGGTTCCGCTACGACTCTG- Wild type Forward; 5′ - CCGGACAAGTTTTTCATCGT - Common Reverse. *Il4ra* ^−/−^ mice were maintained as homozygotes as previously described [18] and genotyped by PCR using primers: 5′ - CCAGACTGCCTTGGGAAAAG - Mutant Forward, 5′ –TGTGGGCTCAGAGTGACAT - Wild type Forward, 5′ - CAGGGAACAGCCCAGAAAAG- Common Reverse. *Sharpin^cpdm-Dem^, Il4ra^−/−^* double mutant mice were generated by intercrossing homozygous male BALB/c-*Il4ra^tm1Sz^*/J mice with heterozygous C.OcB3-*Sharpin^cpdm-Dem^* females. Progeny that genotyped as heterozygous for both alleles were then intercrossed until the *Il4ra^tm1Sz^* allele was fixed to homozygosity. The colony was maintained by mating mice homozygous for the *Il4ra^tm1Sz^* allele and heterozygous for the *Sharpin^cpdm-Dem^* allele. The same strategy was used to generate mice homozygous for the *Rag1^tm1Mom^* allele and heterozygous for the *Sharpin^cpdm-Dem^* allele, which were intercrossed to maintain the colony.

### Phenotyping mutant mice

For each comparison conducted in this study, age matched female and male mutant and control mice were collected, euthanized by CO_2_ asphyxiation, and complete necropsies performed as previously described [47]. Hematoxylin and eosin (H&E) stained slides were examined by experienced board certified veterinary anatomic pathologists (JPS, HH) all lesions subjectively scored (normal, 0; mild, 1; moderate, 2; severe, 3; extreme, 4) and data (with diagnosis and anatomic site) entered into the Mouse Disease Information Database (MoDIS) [48]. These data were used to generate spread sheets for semi-quantitative analysis. In addition, morphometric analyses of tissue sections were done to determine epidermal thickness (dorsal interscapular skin) along the linear length of sample. In each case 10 measurements were made within consecutive 250 um fields along an H&E stained section of dorsal skin from each mouse. Total epidermal thickness (TE, basement membrane to top of the stratum corneum) and thickness of the Malphighian layer (basement membrane to the base of the stratum corneum (BM-SC) were done in vertical sections which were taken in areas meeting the specific critieria of being intrafollicular regions of the epidermis, with the entire length of the hair follicle in the field for consistent orientation. Measurements were done at 400× magnification using a DP70 digital camera on a BH2 photomicroscope (Olympus, Tokyo, Japan) and DP controller 3.2 software (Olympus, Center Valley, PA).

### Hematology

200 ul of whole blood was collected in in heparinized tubes. The uncoagulated blood was run without separation on a Siemens Advia 2120 Hematology Analyzer (Siemens Healthcare Diagnostics Inc; Tarrytown, NY).

### Immunohistochemistry

Cleaved Caspase 3 (CASP3) (Cat#9661, Cell Signalling Technologies; Danvers, MA) and cleaved Caspase 9 (CASP9) (Cat# NB100-56118, Novus Biologicals; Littleton, CO) positive cells were identified by immunohistochemistry in paraffin embedded mouse skin fixed with Fekete's acid alcohol formalin as previously described [49] using a using a Leica Autostainer ST5020 (Leica Microsystems; Buffalo Grove, IL). Counts were made of the number of CASP3 or CASP9 positive cells within a 250 um length of dorsal skin epidermis. Ten measurements were taken from each mouse.

### Quantitative real-time RT-PCR (qRT-PCR)

The expression of *Il4*, *Il5*, *Il13*, *Ifng*, *Ccl11*, *Ccl24*, and *Cxcl10* mRNA in the skin of 4-week old mice was determined by qRT-PCR (Renninger et al., 2005). Skin from 4 weeks of age/sex matched mice was collected and stored in RNALater (Qiagen, Valencia, CA) at −80°C until samples from all replicates were collected. RNA was then extracted using a PureLink RNA Mini Kit (Invitrogen, Grand Island, NY). For each qRT-PCR, a 15 ul reaction was run with 7.5 ul Taqman One-Step RT-PCR Master Mix 2X (Life Technologies, Grand Island, NY), 0.4 ul, Space between number and measurement unit as here or not as in most examples in the paper) 40× Multiscribe and Rnase Inhibitor Mix, 0.75 ul of 20× Assays on Demand Taqman primer and probe set and 100 ng RNA. The qRT-PCR was performed in a Mastercycler® realplex4 (Eppendorf, Hauppauge, NY) programmed at 40 cycles of 42 C for 50 minutes, 90 C for 10 minutes, 95 C for 15 seconds, 60 C for 1 minute, and 72 C for 1 minute. The Ct values for each chemokine were normalized by subtracting the Ct values for the housekeeping gene *Actb* (Delta Ct). The relative fold-change in mRNA expression between wild-type mice and mutant mice was calculated by the 2^−(delta-delta Ct)^ [50].

### Statistical analysis

All analyses were completed using JMP (SAS Institute Inc, Cary NC) linear regression models in lieu of analysis of variance (ANOVA). The effect of genotype was evaluated per phenotype (hematological and skin morphometrics). Prior to testing, each dataset was analyzed to establish conformation with linear regression assumptions. Where necessary, data transformation (i.e., logarithm or square root) was applied to meet assumption criteria. Genotype factor significance (p-value<0.05) indicating that at least two genotype levels differ with respect to their phenotypic measurements was established subject to a Tukey HSD *post-hoc* test (adjusted *p-value*<0.05) to identify significant differences between individual factor levels. Kaplan-Meyer Plots for lifespan were done using n≥5 mice for each genotype based on length of life from birth until The Jackson Laboratory ACUC regulations required euthanasia for morbidity.

## Supporting Information

Figure S1 **Kaplan Meyer plots of lifespan reveals significantly reduced survival rates in** ***Sharpin^cpdm-Dem^*** **,** ***Il4ra*** **^−/−^ mice.** *Sharpin^cpdm-Dem^*, *Il4ra* ^−/−^ mice have reduced average survival when compared to *Sharpin^cpdm-Dem^*, *Rag1^−/−^* compound mutants and to *Sharpin^cpdm-Dem^* or *Sharpin^cpdm^* mutants. *Rag1^−/−^*, *Il4ra* ^−/−^, and WT (*Sharpin^cpdm-Dem^/+*, or *Sharpin^cpdm^/+*) mice all had lifespans exceeding 200 days (Significance indicated by P<0.05).
(TIF)
