# Supplementary figures and images for: Chronic Proliferative Dermatitis in Sharpin Null Mice: Development of an Autoinflammatory Disease in the Absence of B and T Lymphocytes and IL4/IL13 Signaling
Source: PLoS One. 2014 Jan 21;9(1):e85666. doi: 10.1371/journal.pone.0085666 (PMC3897490; doi:10.1371/journal.pone.0085666)

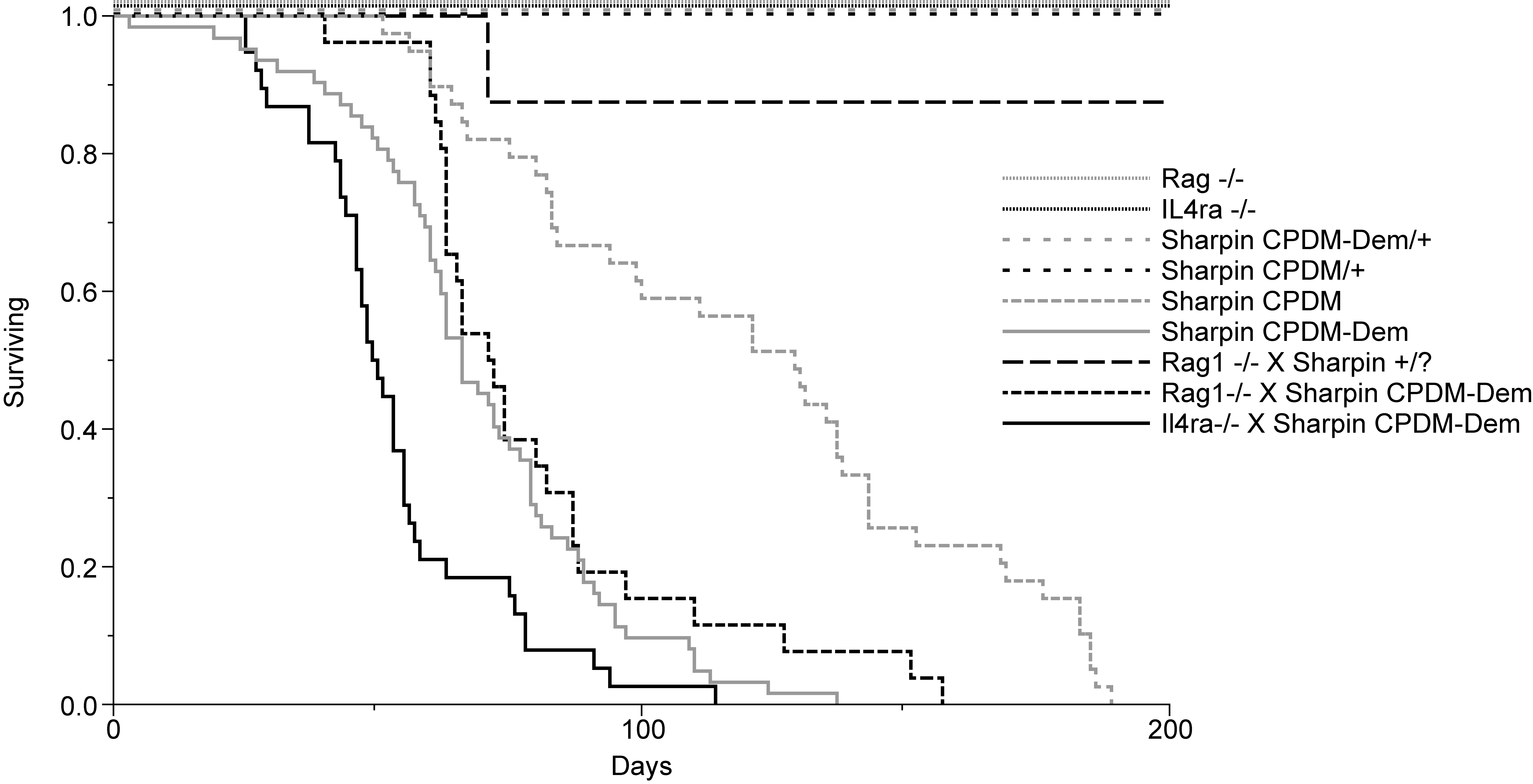

Supplement: Figure S1 — Kaplan Meyer plots of lifespan reveals significantly reduced survival rates in Sharpincpdm-Dem , Il4ra −/− mice. Sharpincpdm-Dem, Il4ra −/− mice have reduced average survival when compared to Sharpincpdm-Dem, Rag1−/− compound mutants and to Sharpincpdm-Dem or Sharpincpdm mutants. Rag1−/−, Il4ra −/−, and WT (Sharpincpdm-Dem/+, or Sharpincpdm/+) mice all had lifespans exceeding 200 days (Significance indicated by P<0.05). (TIF) [file pone.0085666.s001.tif]
